# Key Pathophysiological Role of Skeletal Muscle Disturbance in Post COVID and Myalgic Encephalomyelitis/Chronic Fatigue Syndrome (ME/CFS): Accumulated Evidence

**DOI:** 10.1002/jcsm.13669

**Published:** 2024-12-27

**Authors:** Carmen Scheibenbogen, Klaus J. Wirth

**Affiliations:** ^1^ Institute of Medical Immunology Charité ‐ Universitätsmedizin Berlin, Corporate Member of Freie Universität Berlin and Humboldt Universität zu Berlin and Berlin Institute of Health (BIH) Berlin Germany; ^2^ Mitodicure GmbH Kriftel Germany; ^3^ Institute for General Pharmacology and Toxicology, University Hospital Goethe University Frankfurt am Main Frankfurt Germany

**Keywords:** long COVID, mitochondrial damage, myalgic encephalomyelitis/chronic fatigue syndrome, post–COVID‐19, skeletal muscle, vascular dysfunction

## Abstract

**Background:**

Recent studies provide strong evidence for a key role of skeletal muscle pathophysiology in myalgic encephalomyelitis/chronic fatigue syndrome (ME/CFS). In a 2021 review article on the pathophysiology of ME/CFS, we postulated that hypoperfusion and ischemia can result in excessive sodium and calcium overload in skeletal muscles of ME/CFS patients to cause mitochondrial damage. Since then, experimental evidence has been provided that supports this concept.

**Methods:**

We collect, summarize and discuss the current state of knowledge for the key role of skeletal muscle pathophysiology. We try to explain which risk factors and mechanisms are responsible for a subgroup of patients with post COVID syndrome (PCS) to develop ME/CFS (PC‐ME/CFS).

**Results:**

Mitochondrial dysfunction is a long‐held assumption to explain cardinal symptoms of ME/CFS. However, mitochondrial dysfunction could not be convincingly shown in leukocytes. By contrast, recent studies provide strong evidence for mitochondrial dysfunction in skeletal muscle tissue in ME/CFS. An electron microscopy study could directly show damage of mitochondria in skeletal muscle of ME/CFS patients with a preferential subsarcolemmal localization but not in PCS. Another study shows signs of skeletal muscle damage and regeneration in biopsies taken one day after exercise in PC‐ME/CFS. The simultaneous presence of necroses and signs of regeneration supports the concept of repeated damage. Other studies correlated diminished hand grip strength (HGS) with symptom severity and prognosis. A MRI study showed that intracellular sodium in muscles of ME/CFS patients is elevated and that levels correlate inversely with HGS. This finding corroborates our concept of sodium and consecutive calcium overload as cause of muscular and mitochondrial damage caused by enhanced proton‐sodium exchange due to anaerobic metabolism and diminished activity of the sodium‐potassium‐ATPase. The histological investigations in ME/CFS exclude ischemia by microvascular obstruction, viral presence or immune myositis. The only known exercise‐induced mechanism of damage left is sodium induced calcium overload. If ionic disturbance and mitochondrial dysfunction is severe enough the patient may be captured in a vicious circle. This energy deficit is the most likely cause of exertional intolerance and post exertional malaise and is further aggravated by exertion.

**Conclusion:**

Based on this pathomechanism, future treatment approaches should focus on normalizing the cause of ionic disbalance. Current treatment strategies targeting hypoperfusion have the potential to improve the dysfunction of ion transporters.

## 1 Introduction

Following COVID‐19 infection, a substantial fraction of patients suffers from long‐lasting or persistent symptoms known as long COVID or post–COVID‐19 condition or syndrome (PCS). Symptoms show a broad overlap with myalgic encephalomyelitis/chronic fatigue syndrome (ME/CFS), and a subset of PCS patients fulfils the diagnostic criteria for ME/CFS [1, 2, 3, 4, 5, 6]. ME/CFS is a frequent and debilitating disease triggered by various infections. There is no effective treatment, mainly due to the lack of understanding of the underlying pathomechanisms, which precluded the identification of appropriate targets for drug development. The pandemic has led to more research and initiation of clinical trials. Evidence is now mounting for an involvement of skeletal muscles in the pathophysiology of both conditions.

Skeletal muscle involvement in PCS and ME/CFS is obvious from clinical findings, exercise testing, force measurements, biomarkers, imaging and histological studies. Patients frequently suffer from muscle weakness, pain, fasciculations (involuntary contractions) and cramps. Diminished muscle strength can be shown in most ME/CFS patients by hand grip and quadricep strength measurement [7]. In a prospective observational study, lower hand grip strength early in the disease correlated with symptom persistence in patients with PCS strongly suggesting an important role of skeletal muscle pathophysiology [8]. Exercise testing shows limited exercise capacity, more rapid exhaustion and metabolic changes indicative of anaerobic metabolism and mitochondrial dysfunction that occur early on in exercise as well as reduced oxygen extraction [6, 8, 9, 10, 11, 12, 13, 14]. It is finally clear that exercising skeletal muscles do not produce the necessary amount of energy for enduring performance and that the inability to do so is invariably linked to exercise intolerance and post exertional malaise (PEM), a deterioration of pre‐existing symptoms and a further diminution of exercise and muscle performance compared with pre‐exercise. As exercise intolerance and PEM are the hallmarks of ME/CFS, understanding of the mechanisms involved is key to understanding of ME/CFS pathology, and for the development of effective drugs.

Vascular dysregulation and hypoperfusion are now recognized as central mechanisms for cerebral and muscular disturbance in ME/CFS and PCS [10, 15, 16]. Diminished muscle strength was associated with markers of hypoperfusion in the blood of PC‐ME/CFS patients and with markers of inflammation in PCS [6]. In PCS, many findings show endothelial dysfunction and a capillary‐microvascular perfusion disturbance [17, 18] in which inflammatory capillary wall changes, impaired red blood cell deformability and probably microclots interact with a high vasoconstrictor tone (enhanced vascular constriction) to severely impair microcirculation. This primary microvascular disturbance is strongly aggravated by the diminished cardiac preload [10] resulting in low ventricular filling pressure, low stroke volume and diastolic dysfunction, which has been outlined in a recent publication [19].

The disturbance in skeletal muscle can result not only from diminished perfusion and hypoxemia but also from inflammation or viral infection in skeletal muscle or damage to mitochondria. Both hypoperfusion and impaired mitochondrial function can cause the energetic deficit. Mitochondrial dysfunction is a long‐held assumption to explain cardinal symptoms of ME/CFS. The most convincing evidence has been provided by plasma metabolic studies so far [20, 21].

## 2 Histology of Muscles Provides Evidence for a Predominant Mitochondrial Pathology

Recent EMG and biopsy studies provide strong evidence for the skeletal muscle pathophysiology in PCS and ME/CFS. Pathohistological findings include fibre atrophy, signs of necrosis and regeneration, moderate leukocyte infiltration and capillary rarefication (loss of capillaries) and mostly basement membrane endothelial changes [9, 13, 22, 23, 24].

Two muscle biopsy studies performed in PCS patients with fatigue and exertional intolerance at least 6 months after acute infection with a subset of patients fulfilling diagnostic criteria for ME/CFS deliver the most valuable information regarding vascular and mitochondrial pathology. There were no more signs of capillary obstructions or microclots as reported for the earlier PCS stage [17, 25] and no evidence for viral reactivation in either study as well as only mild signs of inflammation [9, 24]. In the exercise study, nucleocapsid was equally detected in patients as well as in healthy controls with a previous SARS‐CoV2‐infection making a role of the virus unlikely [9]. In the non‐exercise study, SARS‐CoV‐2 RNA could not be detected in the muscle tissues [24]. In patients, fewer capillaries, thicker capillary basement membranes and increased numbers of CD169+ macrophages were found. In the exercise study, the skeletal muscle capillary‐to‐fibre ratio, capillary density and intracellular and circulating lactate concentrations were not different between PCS patients and controls. While in the non‐exercise study patients had been sick less than a year, in the exercise study, they had been sick between 1 and 2 years. Both studies provide evidence of mitochondrial dysfunction, and the exercise study also shows signs of muscle damage with tissue necrosis and more glycolytic fibres [9]. Hence, the initial capillary disturbance is no more seen in those studies [9, 24] but mitochondrial dysfunction and, most interestingly, for the first time necroses as signs of muscle damage in the exercise study. This is most likely due to the fact that the biopsies were optimally timed 1 day after the exercise challenge to detect damage. The finding of necroses emphasizes the role of skeletal muscle pathophysiology for ME/CFS. This study clearly shows that the skeletal muscle damage is related to exercise. In line with this finding, increases of the skeletal muscle proteins tropomyosin TPM4, tropomodulin TMOD3 and calmodulin CALM2 were found in extracellular vesicles and were strongly correlated with higher levels of myalgia after exercise in the ME/CFS patients suggesting at least mild damage [26].

Mitochondrial respiration in skeletal muscle biopsies and metabolomic signatures was indicative of mitochondrial dysfunction [9, 13, 24]. A recent study performing mRNA sequencing from muscle biopsies provides further evidence of diminished mitochondrial function [27]. Electron microscopy revealed mitochondrial damage in muscles in ME/CFS [13]. Subsarcolemmal mitochondria were preferentially damaged compared with interfibrillary mitochondria. Table 1 gives an overview of the results of the skeletal muscle biopsy studies.

**TABLE 1:** Overview of skeletal muscle biopsy studies in ME/CFS and PCS.

Study	Patients	Type of study	Findings
Appelman et al. [9]	PCS patients (*n* = 25) 1–2 years postinfection. All with PEM by the DSQ‐PEM criteria	● Light microscopic investigation before and after exercise (ergometry)‐inducing PEM ● In vitro mitochondrial function	Reduced exercise capacity, metabolic changes in vitro indicative of mitochondrial dysfunction. Amyloid depositions outside capillaries increasing by exercise and focal fibre atrophy, more glycolytic fibres, moderate leukocyte infiltration, focal necrosis, which increased significantly after exercise. Signs of regeneration and mild damage in biopsies before and after exercise.
Bizjak et al. [13]	PCS patients (*n* = 13) with a median of 15 months postinfection. ME/CFS patients (*n* = 15) fulfilling CCC criteria with a median of 23 months postinfection.	● Light and electron microscopy ● Ergometry ● In vitro mitochondrial function	Reduced exercise capacity and mitochondrial dysfunction in vitro but no morphological mitochondrial changes compared with healthy controls. Reduced exercise capacity and mitochondrial dysfunction in vitro. Mitochondria show detrimental alterations of several parameters of mitochondrial morphology with a preferential localization in the subsarcolemmal area in contrast to interfibrillary mitochondria.
Aschman et al. [24]	PCS and PCS‐ME/CFS (CCC) patients (*n* = 11) 6 months to 1 year postinfection.	● Light and electron microscopy ● MRI ● Muscle proteomics	Muscle weakness. Type‐2b‐fibre atrophy and increased numbers of tissue macrophages. Decreased capillary‐to‐fibre ratio and increased capillary basement membrane thickness. No signs of myositis. Decreased expression of genes related to mitochondrial activity.
Hejbøl et al. [22]	PCS patients (*n* = 16) 5–14 months postinfection	● Light and electron microscopy ● Electromyography	Muscle weakness and myopathic electromyography. Muscle fibre atrophy, indications of fibre regeneration. Mitochondrial changes, comprising loss of cytochrome c oxidase activity, subsarcolemmal accumulation and/or abnormal cristae. Inflammation as T lymphocytes and/or muscle fibre human leukocyte antigen I expression. Capillaries affected, involving basal lamina and cells.
Agergaard et al. [23]	PCS patients (*n* = 8) median 9.8 months postinfection	● Light and electron microscopy ● Electromyography	Muscle strength reduced. Myopathic changes in quantitative EMG or abnormal single fibre EMG or both. Microstructural damage to terminal motor nerves and motor endplate.
Walitt et al. [27]	ME/CFS patients (*n* = 8–17) median 33 ± 15 months postinfection	● RNA sequencing ● Mitochondrial genome ● Mitochondrial function	Males: downregulated mitochondrial and hexose metabolism, upregulated fatty acid beta oxidation. Female: downregulated mitochondrial and fatty acid metabolism. No difference in mitochondrial function of blood immune cells.

Thus, in all these studies, neither viral persistence, overt inflammation, myositis nor obstructed capillaries are found as the cause of muscle damage, but mitochondrial damage or dysfunction could be shown. Electronic microscopy revealed the damage of mitochondria in skeletal muscle in PC‐ME/CFS but not in PCS patients [13]. The absence of obstructed capillaries does not mean that perfusion is normal; it could be impaired by functional mechanisms such as endothelial dysfunction, high vasoconstrictor tone or autoantibodies against vasoregulatory receptors [28, 29]. It is important to note that these considerations apply to PCS with fatigue, exertion intolerance and PEM or ME/CFS, and not to other subsets of PCS where other mechanisms may still be operative. Studies of circulating miRNAs in ME/CFS provide further evidence for impairments in exercise hyperaemia, angiogenic adaptations to hypoxia, antioxidant defences and mitochondrial function [28, 29]. Potential mechanisms involved in skeletal muscle pathophysiology in ME/CFS have also been highlighted in a recent review paper on ME/CFS [30]. Altogether, long suspected skeletal muscle pathology, tissue damage and mitochondrial damage and dysfunction have now been clearly demonstrated by directly analysing muscle tissue in ME/CFS. Table 2 gives an overview of the accumulated evidence of skeletal muscle involvement arranged according to the investigational method.

**TABLE 2:** Skeletal muscle pathological findings in patients with PCS with PEM or ME/CFS.

**Clinical signs:** ● Fatigue, early exhaustion, muscle weakness, muscle pain, fasciculations and cramps [6, 7, 8]
**Skeletal muscle force test:** ● Muscular force impaired assessed via hand grip strength (HGS) and leg strength [7]
○ HGS correlates with symptom severity and prognosis
**Exercise (endurance) test:** ● Metabolic changes during exercise: early appearance of anaerobic metabolism [9, 10, 31]
● Diminished oxygen uptake and extraction [9, 10, 14, 31]
**Biopsies from skeletal muscle:** ● Skeletal muscle biopsies show signs of muscular damage and regeneration. More damage 1 day after exercise [9]
○ Biopsies exclude obstructed capillaries, viral presence and autoimmune myositis
● Evidence for muscle mitochondrial dysfunction [9, 13, 22, 24, 27]
● Electron microscopy from skeletal muscle biopsies shows changes in mitochondrial morphology and mitochondrial damage [13, 22]
○ Mitochondrial damage shows a particular localization pattern. Subsarcolemmal mitochondria are affected in contrast to interfibrillary mitochondria
**Imaging (MRI):** ● Intracellular sodium in skeletal muscle is elevated and negatively correlates with handgrip strength in a 23‐Na‐MRI study in resting or exercising muscle depending on muscle type [12]
○ Rise in intracellular sodium is a precondition for damaging calcium overload

## 3 Sodium and Consecutive Calcium Overload as the Cause of Mitochondrial Pathology in ME/CFS

After having summarized the recent evidence for skeletal muscle pathology and mitochondrial dysfunction, we address the possible pathomechanisms. The recent electron microscopic investigations show alterations in mitochondrial morphology in ME/CFS finally proving mitochondrial damage [13]. This study, however, does not reveal the damaging mechanisms. Possible mechanisms of mitochondrial damage discussed include inflammation, viral infection, autoantibodies and damage by calcium overload. The latter can be caused by muscular hypoperfusion upon exertion and consecutive dysregulation of ion transport resulting in proton and consecutive sodium and calcium overload, which has been outlined in our previous papers and summarized in Table 3 [19, 31].

**TABLE 3:** Disturbed sodium and calcium homeostasis resulting from microvascular disturbances can cause mitochondrial toxicity [19, 31].

● Hypoperfusion during exercise causes anaerobic metabolism raising proton production ● The sodium‐proton‐exchanger subtype 1 (NHE1) extrudes the protons via an import of sodium (1:1) leading to sodium rise ● The activity of the Na^+^/K^+^‐ATPase is insufficient due to lack of ATP, inhibition by mitochondrial ROS, dysfunction of ß2 adrenergic receptors (autoantibodies, stress‐mediated desensitization) and calcitonin‐gene–related peptide (CGRP) shortage (due to small fibre neuropathy) to remove high sodium loading causing further sodium overload ● The sodium‐calcium exchanger (NCX) changes into reverse transport mode importing calcium instead of exporting it ● The ensuing calcium overload affects the mitochondria and cytoplasmatic metabolism

The skeletal muscle damage found in ME/CFS was shown to be related to exercise [9]. This is in line with a recent study showing exercise‐induced increase of markers related to muscle damage in peripheral blood extracellular vesicles [26]. Based on our current knowledge on the known causes of muscle damage related to exercise and malperfusion, diminished function of ion transporters and consecutive calcium overload‐induced toxicity is the only explanation for tissue necroses and particularly mitochondrial damage in ME/CFS. Calcium overload occurs as a consequence of excessive sodium loading in myocytes. Elevated intracellular sodium levels in skeletal muscle could indeed been shown in an MRI study with 23‐Na^+^ in ME/CFS patients [12]. Depending on the muscle type, sodium was already elevated before exercise or was found elevated after exercise. Calcium overload cannot be demonstrated in vivo by imaging techniques, but the histological picture found in the biopsy study after exercise showing necroses can only be explained by calcium overload [9]. This histological picture found is reminiscent of ischemia–reperfusion experiments and toxicological studies with high doses of drugs that raise calcium in the heart (unpublished own data). Calcium overload and the consecutive impaired energy metabolism can well explain the exercise intolerance and PEM in ME/CFS. Muscle cramps and fasciculations can occur in ME/CFS already upon minor exertions and can be explained by calcium overload, too. As calcium in the muscle cell is about 10^4^ times lower than the ionic calcium concentration outside the cell membrane [32], cellular calcium overload does not result in changes in plasma calcium.

The electron microscopic study showed a particular distribution pattern with damage to *subscarcolemmal* but not interfibrillary mitochondria in ME/CFS [13] suggesting proximity to a damaging mechanism. In this subscarcolemmal location close to the cell membrane, where the NCX is located, calcium concentration must be highest after the NCX has turned into the reverse mode at high intracellular sodium to import calcium causing mitochondrial damage by calcium overload. Such mitochondrial damage was not seen in PCS in this study [13]. Mitochondrial changes, comprising loss of cytochrome c oxidase activity, subsarcolemmal accumulation and/or abnormal cristae, were seen in PCS patients where it is not clear whether they fulfilled the criteria for ME/CFS [22]. Thus, after SARS‐CoV‐2 infection, a presumed dominant capillary or microvascular disturbance in PCS can cause mitochondrial damage and dysfunction in a subset of patients. Mitochondrial dysfunction in muscles can maintain itself and aggravate or induce a new type of dysfunctional vascular perfusion while the original capillary damage or microvascular disturbance can heal or improve to a considerable extent. Additional risk factors for vascular dysfunction probably cause the transition from PCS to PCS‐ME/CFS as outlined below. In contrast to skeletal muscle, studies of mitochondrial function of peripheral blood immune cells of ME/CFS patients have shown inconsistent results [27, 33, 34].

## 4 The Mechanisms for the Persistence of Mitochondrial Dysfunction and Chronification of ME/CFS

Once mitochondrial dysfunction in skeletal muscle is fully developed, it can be self‐maintaining, causing a new type of vascular disturbance strongly driven by reactive oxygen species (ROS) [19, 31, 35, 36]. Mitochondrial dysfunction not only causes vascular dysfunction but also favours itself as low ATP and ROS inhibit Na^+^/K^+^‐ATPase [37]. This can aggravate sodium and subsequent calcium overload and damage. Hypovolaemia evolving in ME/CFS causes orthostatic stress to desensitize ß2 adrenergic receptors, which are important for the activation of the Na^+^/K^+^‐ATPase [38, 39].

The increased anaerobic metabolism as proven by the higher number of glycolytic fibres [9] due to mitochondrial dysfunction and malperfusion causes an excessive proton generation and consecutive sodium overload so that already, small efforts can lead to calcium overload, calcium‐induced tissue necroses and mitochondrial damage. Signs of regenerating muscle fibres in the skeletal muscle biopsy in parallel with signs of recent necroses and some necroses present in the biopsy already before exercise testing strongly suggest that repeated damaging events have taken place [9]. Such a repetitive nature of the damage can be well explained by increased intracellular sodium in myocytes being or quickly getting close to the reverse mode threshold of the NCX so that otherwise non‐damaging exercise causes skeletal muscle and mitochondrial damage [31]. Through this strong tendency to develop PEM upon minor exertion and the repetitive damage by small efforts (low PEM threshold), the disease can maintain itself to finally become independent of the original triggers and chronic.

Our hypothetical disease mechanism implies that damage and regeneration occur in parallel in skeletal muscle as was proven by histology of ME/CFS patient muscle biopsies [9]. At a certain point, the number of intact mitochondria able to replicate for the full repletion of the pool of intact mitochondria is probably too low. The lower the pool of intact mitochondria is the more anaerobic metabolism prevails. The increase in the number of glycolytic fibres found in the biopsies may be seen as an adaptive mechanism [9]. Anaerobic metabolism leads to a higher proton load and, by that, a higher sodium load favouring calcium overload and damage. This is a possible vicious circle that could develop once disease has progressed and can explain the misery of the most severe and bedridden patients. Investigations on the state of the mitochondria by electron microscopy ranging from healthy people to mild to moderate and severe cases would be therefore highly desirable.

**FIGURE 1:**
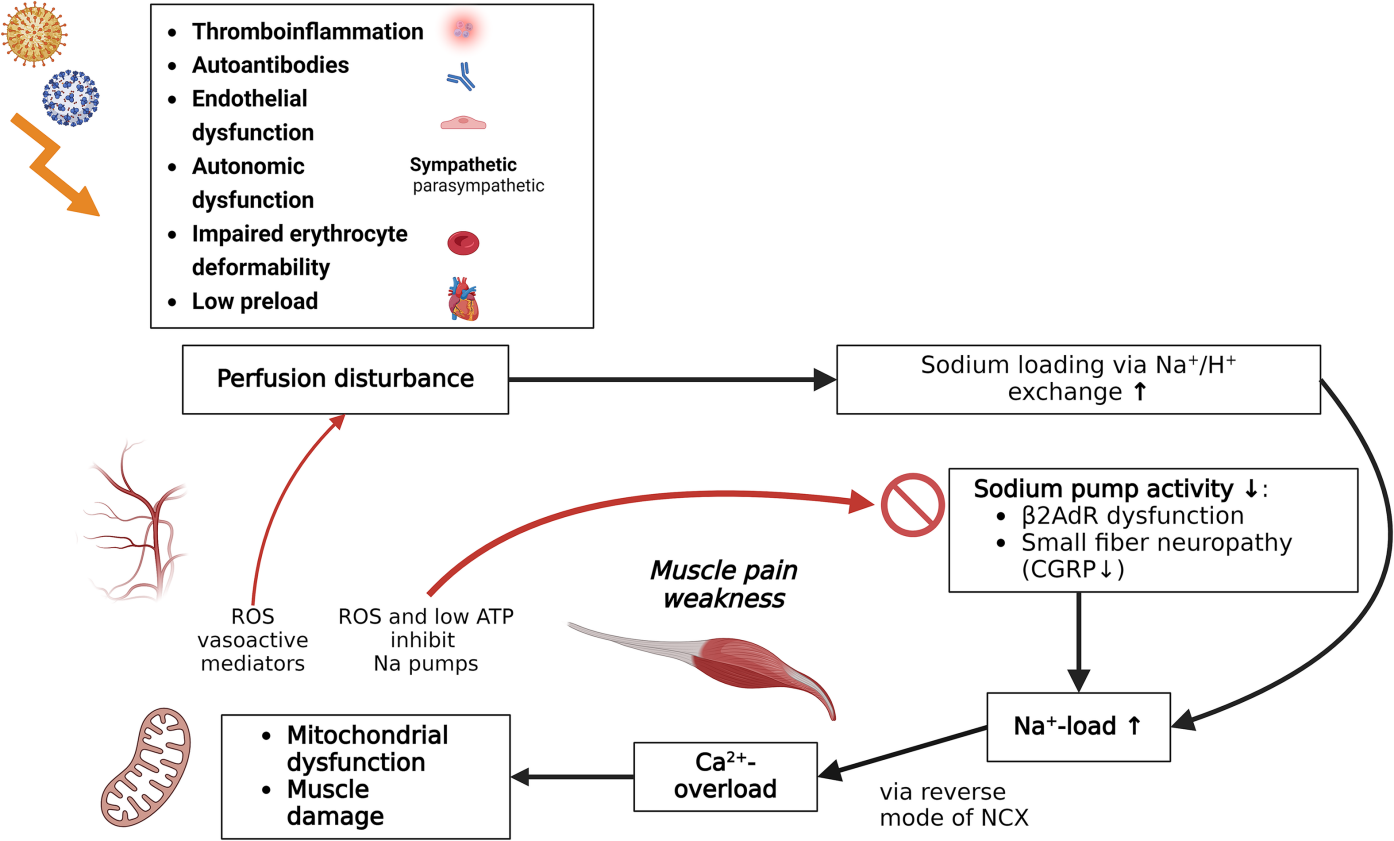
Several mechanisms triggered by an infection can result in circulatory disturbance and hypoperfusion of muscles including autoantibodies [41], inflammation [22], sympathetic overactivity [10], endothelial dysfunction [42], impaired red blood cell deformability [43] and diminished preload [10]. Upon exertion, hypoperfusion of skeletal muscles leads to rise in protons, intracellular sodium and calcium [12], muscle necrosis [9], mitochondrial damage and enhanced production of reactive oxygens [13, 22]. In ME/CFS, the activation of the sodium pump (Na^+^/K^+^‐ATPase) upon exertion may be diminished due to low ATP and dysfunctional ß2AdR and CGRP. At a certain level of intracellular sodium, the sodium‐calcium‐exchanger NCX changes its transport mode from calcium export to calcium import causing calcium overload that causes mitochondrial and myocyte damage. As a result, low ATP and ROS production further impair the sodium pump. ROS also impairs vascular function and perfusion. A vicious circle arises. ß2AdR: ß2‐adrenergic receptor; CGRP: calcitonin‐gene–related peptide; ROS: reactive oxygen species. Biorender was used to create the figure.

Altogether, we assume that mitochondrial dysfunction that triggers and reproduces itself in skeletal muscle at low levels of effort does constitute the central pathomechanism of ME/CFS. Thus, although ME/CFS has different triggers and may have different causes [40], these finally can lead to and converge to the same pathomechanism carried on by vascular disturbances and secondary mitochondrial dysfunction. Thus, one could consider ME/CFS as an acquired ischemic mitochondrial myopathy (AIMM). The development of self‐maintaining mitochondrial dysfunction out of primary vascular disturbances is shown in Figure 1. How the poor energetic situation in skeletal muscle then can affect cardiovascular function apart from the generation of ROS to impair perfusion and to cause hypovolaemia and other symptoms and systemic sequelae typical of ME/CFS has been explained in detail in previous publications [38, 44].

In addition to potential vascular disturbances by ROS, cardiovascular disturbances like hypovolaemia, low ventricular filling pressure, endothelial dysfunction, high sympathetic tone and autoantibodies to vasoregulatory receptors, which have all been reported for ME/CFS (for review [19]), may aggravate the vascular dysfunction in ME/CFS as shown in Figure 1 [38]. There is also evidence of reduced erythrocyte deformability impairing their capillary flow [43]. The rarefication of capillaries reported in two studies can certainly contribute to malperfusion [24, 45]. Microclots may disappear, and capillary wall damage may heal, but residual damage cannot be excluded. The question is whether capillary rarefication or basement membrane thickening observed in patients less than 1 year after COVID is a residual damage or a part of the new vascular pathology [9, 22, 23, 24, 45]. It should also be considered that a transition state must exist that may confuse the picture.

The considerations made above imply that the transition to or development of severe mitochondrial dysfunction in skeletal muscle can cause severe PEM and thus ME/CFS developing in PCS and can most likely be generalized for ME/CFS of other causes. A probably initially predominant capillary‐microvascular disturbance is shifted into a mitochondrial‐vascular disturbance and explains why the disease cannot heal or even aggravates due to this self‐perpetuating mechanism as outlined below.

## 5 Transition of PCS to ME/CFS and Risk Factors

A yet unresolved question is why only a subset of PCS patients with fatigue and exertional intolerance develop ME/CFS with the characteristic of more severe and longer lasting PEM [6]. While symptoms in PCS not fulfilling ME/CFS criteria can improve over the course of 1–2 years, symptoms persist or even aggravate in most ME/CFS patients [1, 8]. Thus, the question arises if ME/CFS simply is a severe and persistent form of PCS or if other or additional pathomechanisms become operative behind a clinical picture with similar symptoms in the transition from PCS to ME/CFS. In other words, has something changed ‘behind the scene’?

Microvascular impairment later in disease course may be more of a functional nature in which endothelial dysfunction and ROS generated from mitochondrial dysfunction and probably autoantibodies to vasoregulatory receptors play a role, while in the early stage, impaired capillary microcirculation as a result of the combined effects of inflammatory capillary wall changes and pathological blood components may be the predominant disturbance [17, 18, 25, 42, 46, 47, 48, 49]. The latter are no more found in the above referenced histological studies in PC‐ME/CFS [9]. Besides this change in the vascular pathomechanisms over time, a difference can also be seen in the mitochondrial pathology between PCS and ME/CFS. In ME/CFS, mitochondria were morphologically altered and damaged, preferentially in the subsarcolemmal area while no such damage was seen in PCS [13].

Understanding why a transition to the development of severe mitochondrial dysfunction and damage occurs to prevent healing and to develop ME/CFS and what the risk factors are is of outmost importance. Autoimmunity and autoantibodies may be strongly involved [41, 50, 51, 52]. We and others found autoantibodies to G protein‐coupled receptors (GPCRs) to be associated with symptom severity in PCS [41, 50, 53]. In our study fatigue, cognitive function and impaired microcirculation correlated with GPCR autoantibodies predominantly in PCS fulfilling ME/CFS criteria [41]. These results are in line with previous findings in post‐infectious ME/CFS patients, which described correlations between clinical symptoms, structural central nervous system (CNS) alterations and levels of autoantibodies against adrenergic receptors and other GPCR [54, 55, 56].

**FIGURE 2:**
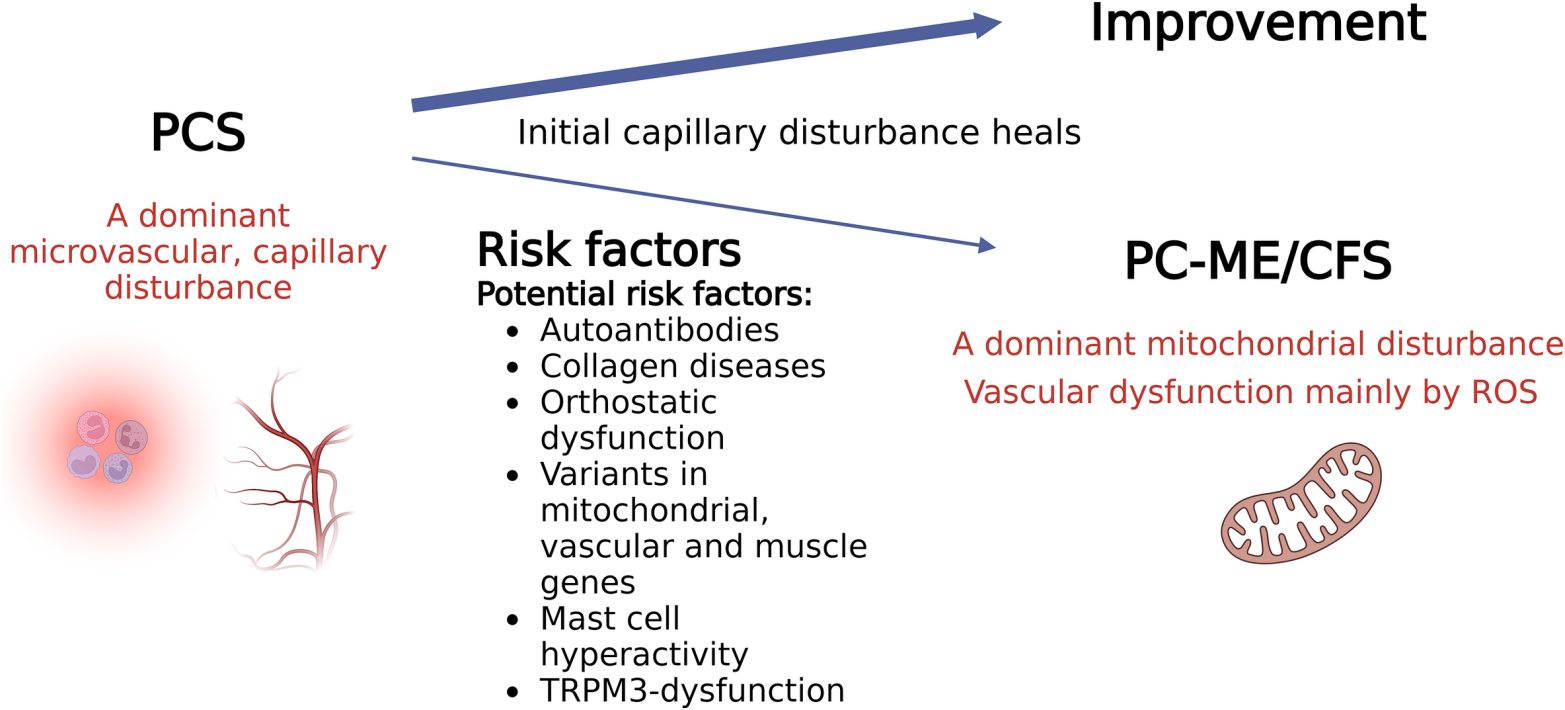
In PCS with fatigue and exertional intolerance, a predominant microvascular disturbance with inflammation was found early in disease [17, 25] which was no more evident later [9, 24]. While patients with PCS frequently improve in health during the 2nd year, those with PC‐ME/CFS stagnated at their initial level [8]. Risk factors for the development of ME/CFS are autoantibodies, collagen diseases like Ehlers–Danlos and joint hypermobility as well as genes regulating mitochondrial, vascular and muscle function [59, 60]. Mast cell hyperactivity and dysfunction of the ion channel TRPM3 may be the other risk factors [61, 62, 63].

There is evidence for differences in the vascular pathologies, symptoms, findings and their correlations between PCS and PC‐ME/CFS that suggest a transition takes place. Biomarkers for vascular function and angiogenesis showed considerable differences [42, 57]. Sera from PC‐ME/CFS patients had increased anti‐endothelial cell autoantibodies and differed in their functional effects on endothelial cells, i.e., secretion profiles and angiogenic potential [57]. PCS sera enhanced the release of molecules associated with vascular remodelling and significantly promoted angiogenesis in vitro compared to the PC‐ME/CFS. A pro‐angiogenic effect of PCS sera as a compensatory mechanism to endothelial dysfunction was assumed, which is absent in PC‐ME/CFS patients. In another study comparing PCS with PC‐ME/CFS patients, endothelial dysfunction and elevated endothelin‐1 levels were found in PCS and PC‐ME/CFS patients, indicating that hypoperfusion plays a role [42]. However, a paradoxical association of the reactive hyperaemia index with age, blood pressure and BMI as well as diminished Ang‐2 in the PCS group was considered suggestive of distinct vascular pathomechanisms between both conditions. Retinal microvasculature image analysis using optical coherence tomography angiography clearly showed retinal vascular damage 138 days after acute infection of hospitalized patients [46]. This investigational method enables detailed analysis of this accessible microvasculature bed that is known to have homology with systemic vasculature. By contrast, in patients with PCS 15 months on average after SARS‐Cov‐2 infection, there were no long‐term structural signs of damage in the retina [58]. Although it is not clear, whether these findings were accompanied by clinical improvement, there were no more pathological findings in the retinal vessels, implying that the microvascular disturbance heals. It is important to note that the vascular disturbances in PCS usually affect the systemic vasculature while exercise‐triggered mitochondrial dysfunction primarily affects the skeletal muscle. ROS levels produced by the dysfunctional mitochondria are then highest in skeletal muscle. These findings support the assumption of a shift in the vascular pathology over time (Figure 2). Hence, not only is there a change in mitochondrial pathology but also in vascular pathology in the transition from PCS to ME/CFS. Finally, genetic risk factors most likely play a role. Ehlers–Danlos and joint hypermobility are risk factors for ME/CFS and are associated with vascular diseases [28]. Genetic risk factors for severe and fatigue‐dominant long COVID and commonalities with ME/CFS were identified by combinatorial analysis including genes regulating mitochondrial, vascular and muscle function [59, 60]. A recent study analysing genomic DNA from muscle mitochondria of ME/CFS patients showed that all patients had variants in mitochondrial genes, however of uncertain significance [27]. Mast cell hyperactivity and dysfunction of the ion channel TRPM3 may be the other risk factors [61, 62, 63]. Figure 2 shows the concept for transition from PCS to ME/CFS and potential risk factors.

## 6 Conclusion and Therapeutic Outlook

Altogether, recent studies investigating skeletal muscle pathology have led to an enormous progress in understanding the pathophysiology of ME/CFS. Skeletal muscle and mitochondrial damage are finally proven and can explain exertional intolerance and PEM. The diminished skeletal muscle force as a biomarker of muscle damage correlates with other key symptoms providing evidence that it is central in the pathomechanism. A self‐reproducing mitochondrial dysfunction most likely constitutes the final and common disturbance of ME/CFS, which locks the patients in a vicious circle from which they can hardly escape. As the disease is self‐maintaining at this stage, treatment of the original trigger, e.g., a persistent virus or vascular inflammation, does most likely not lead to a therapeutic success.

Currently, treatment strategies are pursued to improve vascular perfusion. The inhibition of the acetylcholine esterase by Mestinon^R^ has the potential to improve perfusion by enhancing preload and cardiac output [64]. The guanylate cyclase activator vericiguat induces vasodilation via directly stimulating the vasodilator cGMP in a nitric oxide mimetic manner (NCT05697640) [65]. Rise in cGMP can also improve erythrocyte deformability [66, 67].

Another promising approach is the depletion or neutralization of autoantibodies targeting GPCR vasoregulatory receptors. Immunoadsorption, which can transiently remove autoantibodies, was shown to improve symptoms in two thirds of patients and normalize diminished endothelial function in observational studies [68, 69, 70]. Novel treatments efficiently targeting autoantibody‐producing B cells or plasma cells can achieve an effective and long‐lasting depletion of autoantibodies, and first studies are already ongoing [71]. An innovative concept neutralizing GPCR autoantibodies with an aptamer is currently tested in a multicentre phase II trial (NCT05911009). However, autoantibodies probably do not play a role in all ME/CFS and PCS patients.

ME/CFS is no more an enigmatic disease for which therapeutic concepts are missing. Since the assumed disturbances are functional in nature and are treatable by appropriate agents, there is a good chance of novel highly efficacious drugs and even healing for this frequent and most debilitating disease. We appeal to politicians, pharmaceutical companies and stakeholders to support the rapid development of such promising new drugs.

## Ethics Statement

The authors have nothing to report.

## Conflicts of Interest

Charité holds patents for the use of beta‐adrenergic receptor antibodies in the diagnosis of CFS and for soluble guanylate cyclase activators for treating chronic vascular dysfunction. Carmen Scheibenbogen received honoraria from Celltrend, Roche and Bayer for consultation.

Klaus J. Wirth is the managing director of Mitodicure GmbH, a startup developing a small molecule therapeutic for the treatment of ME/CFS.
